# Public health nurse educators’ conceptualisation of public health as a strategy to reduce health inequalities: a qualitative study

**DOI:** 10.1186/s12939-015-0146-2

**Published:** 2015-02-03

**Authors:** Mzwandile A Mabhala

**Affiliations:** Department of Community Health and Wellbeing, University of Chester, Riverside Campus, Chester, CH1 1SF UK

**Keywords:** Social justice, Inequalities in health, Public health, Socioeconomic determinants, Nursing

## Abstract

**Background:**

Nurses have long been identified as key contributors to strategies to reduce health inequalities. However, health inequalities are increasing in the UK despite policy measures put in place to reduce them. This raises questions about: convergence between policy makers’ and nurses’ understanding of how inequalities in health are created and sustained and educational preparation for the role as contributors in reducing health inequalities.

**Aim:**

The aim of this qualitative research project is to determine public health nurse educators’ understanding of public health as a strategy to reduce health inequalities.

**Method:**

26 semi-structured interviews were conducted with higher education institution-based public health nurse educators.

**Findings:**

Public health nurse educators described health inequalities as the foundation on which a public health framework should be built. Two distinct views emerged of how health inequalities should be tackled: some proposed a population approach focusing on upstream preventive strategies, whilst others proposed behavioural approaches focusing on empowering vulnerable individuals to improve their own health.

**Conclusion:**

Despite upstream interventions to reduce inequalities in health being proved to have more leverage than individual behavioural interventions in tackling the fundamental causes of health inequalities, some nurses have a better understanding of individual interventions than take population approaches.

## Background

Since the publication of the *Independent inquiry into inequalities in health report* [1] reducing inequalities in health has been a benchmark for all UK health and social care policies. Several policy documents identified nurses as key contributors to strategies to reduce health inequalities [2-7]. The evidence suggests that although policy measures to reduce inequalities resulted in an overall improvement in the health of the UK general population, inequalities are widening [8-12]. This raises questions about: convergence between policy makers’ and nurses’ understanding of how the inequalities in health are created, sustained and educational preparation for the role as contributors in reducing health inequalities. Furthermore, the lack of a consistent and ideologically bounded strategy to tackle health inequalities has made the concept malleable by proponents of contrasting interventionist and non-interventionist ideologies. Interventionists’ views of inequalities in health favour upstream population-based activities associated with tackling core determinants of health inequalities, while non-interventionists favour activities associated with encouraging individuals to make healthier choices and take responsibility for their own health [13].

In this paper ‘inequalities in health’ refers to uneven distributions of health benefits and disease burdens that are unjust, unfair and avoidable [13,14]. It is believed that tackling health inequalities is a matter of social justice [15-20] – the idea of creating a society with social institutions based on principles of equality and solidarity, that understand and value human rights, and that recognise the dignity of every human being [19]. As Rawls [21] proposed, fairness, justice and equality are key attributes of social justice.

### Conceptualisation of the inequalities in health

**Figure 1:**
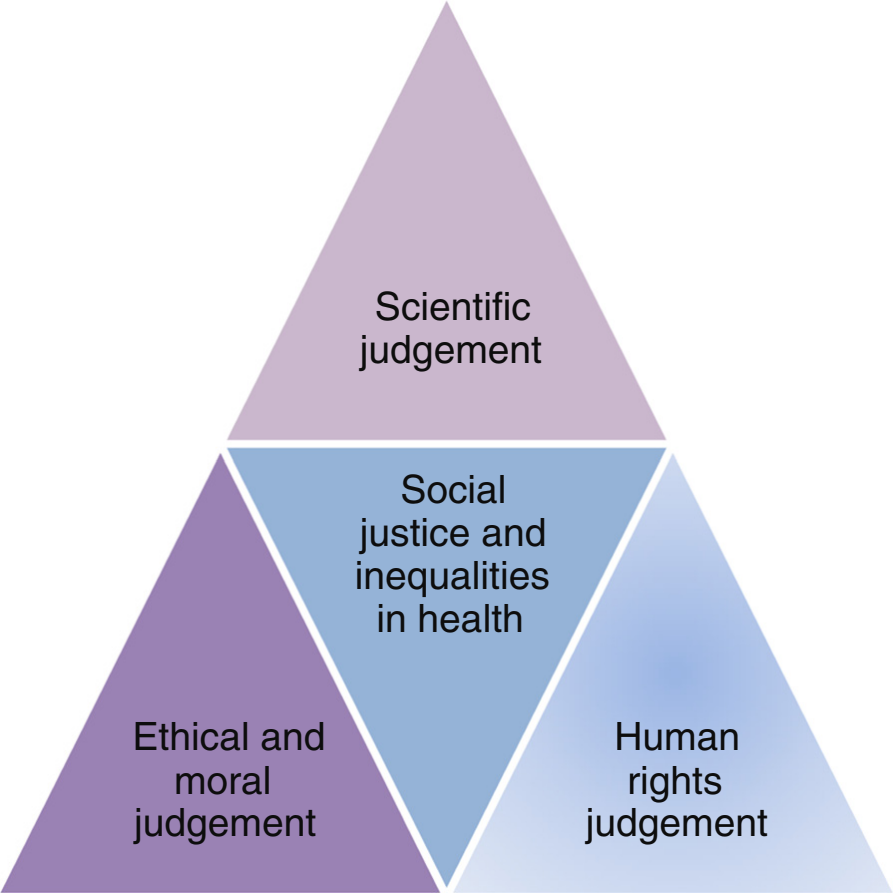
**The triad model of the inequalities in health.**

In this paper seeing reducing health inequalities as a matter of social justice stems from evidence that too many people die prematurely due to uneven distribution of the determinants of health [14]. Mabhala argued that avoidable premature loss of life is more than a matter of statistical evidence: besides being a moral and ethical concern, where it is possible to provide social goods essential for the support of health and well-being and these are not provided, then that is a human rights issue [14]. He therefore proposed that health inequalities can be conceptualized in three dimensions. Figure 1 illustrates a triad model of social justice to frame the argument about health inequalities.

The science dimension enables us to establish evidence of the association between disease and social environment, and explain the pattern and distribution of disease and health [1,22-24]. Through this knowledge we can demonstrate that the distribution of disease follows a social class gradient, and argue that socially produced diseases are avoidable [12,23].

The ethical and moral dimension is the view that socially produced diseases are unfair and unjust, and that tackling them is the right thing to do [20]. As Marmot argued, where systematic inequalities in health are avoidable and are not avoided, then they are unfair, and taking action to put them right is a matter of social justice [20].

The human rights dimension is based on the Alma-Ata declaration of health as a human right [25]; this affirmation aimed to bring concern for improving the health of the disadvantaged from the voluntary realm of charity to the realms of law and entitlement [26].

### Developing the public health nursing role

In the UK the nurses’ role in tackling health inequalities was first made explicit in 2001 in the Annual Report of the Chief Medical Officer [5]. This identified three major categories in the public health workforce: specialist (people who work at senior strategic and policy level e.g. public health directors, public health consultants etc.), practitioner (people who conduct operational, face-to-face public health work e.g. public health nurses, public health managers etc.) and wider workforce (who have or are developing a public health remit as part of their role). It placed the majority of nurses within the practitioner category. Following this, health professional bodies developed competence benchmarks in line with these categories. For example, in 2004 the UK Faculty of Public Health developed a multidisciplinary *National occupational standard for the practice of public health guide* [27], which identified ten key areas of public health specialists’ competence. Based on this several professional development programmes were created, mostly aimed at public health specialists; very little or nothing was produced for the practitioners and wider public health workforce categories.

In response the UK Nursing and Midwifery Council [28] developed standards for specialist community public health nurses. This led to the development of educational programmes aimed at nurses who aspire to gain that status. The UK Department of Health recognised that there were large public health workforces unaffiliated with professional regulatory bodies who were being left out of these developments, and in 2005 commissioned Public Health Resource Unit and Skills for Health to develop a ‘public health skills and career framework as a tool for describing the skills and knowledge needed across all the multidisciplinary groups and levels of public health workforce’ [29]. This framework makes a distinction between four core and five non-core areas of public health practice. The core areas were: 1) surveillance and assessment of the population’s health and wellbeing; 2) assessing the evidence of effectiveness of intervention programmes and services; 3) policy and strategy development and implementation; and 4) leadership and collaborative working. The non-core areas identified were: 1) health improvement; 2) health protection; 3) public health intelligence; 4) academic public health; and 5) health and social care quality [29]. This framework defines nine levels of competence and knowledge, ranging from level one (those with minimal knowledge and skills) to level nine (those with extensive expertise in public health) [29]. These developments helped to identify levels at which nurses are expected to function within public health professions; however, it remained unclear what nurses are actually required to do to reduce inequalities in health.

Several writers attempted to describe the nature and levels of contribution of nurses and other multi-disciplinary health professionals to reducing health inequalities [30,31]. For example Grumbach, Miller, Mertz & Finocchio [31] describe three levels of intervention to reduce inequalities: 1) reducing an unfair distribution of determinants of health inequalities; 2) reducing the unfair distribution of healthcare provision; and 3) assisting individuals to overcome avoidable health inequalities. Mackenbach [30] proposes that health professionals should think of their interventions in terms of an imaginary ‘ladder of political activism’ with four rungs. The first or lowest rung is political passivism – that is, information on health risks and opportunities for health improvement are exchanged within the health sector only, and politicians are only informed if they ask for it. On the second rung, public health professionals actively disseminate relevant information among politicians, for example by addressing their reports to the government, by drawing the attention of the media, and by participating in advisory committees. On the third rung public health professionals may try to directly influence the political process, for example by lobbying and by actively engaging politicians of specific political parties. On the highest or fourth rung, public health professionals become politicians themselves, trying to obtain positions in government or parliament to reach their objectives. Marmot [32] identifies five areas where doctors can help reduce inequalities: 1) work with individual patients, their families and contacts, using clinical tools including social prescribing and brief interventions; 2) work with communities, for example by commissioning measures; 3) use evidence and influence to have a positive impact on health inequalities; 4) use expertise to advocate for change outside traditional medical areas; and 5) promote the generation of research. These frameworks support Mackenbach’s [24] position that reducing health inequalities requires a combination of downstream, midstream and upstream interventions.

Despite upstream interventions to reduce health inequalities being proved to have more leverage than individual behavioural interventions when tackling fundamental causes [12,20,24,31,33,34], the evidence shows nurses are likely to perform individual-family level interventions rather than population approaches [35]. For example, some UK studies found that contrary to the government’s endorsement of their public health role, nurses spend a substantial proportion (61%) of their time on intervention at individual levels [35-38]. One of the explanations for this is that their capacity to undertake public health work is constrained by workload pressures and competing priorities [35]. These findings are consistent with some studies from outside the UK; for example, Grumbach et al. [31,39] suggest that the population health focus is not reflected in practice activities or educational preparation for public health nurses.

Studies have reported that community public health nurses have limited knowledge and skills in promoting upstream, population-based public health interventions [36-38]. The lack of an upstream focus by nurses has been attributed to them being better educated in individual interventions than system interventions [35,40]. For example, participants in Cameron and Christie’s [35] research reported that there was little social science theory or practical public health skills training within their course curricula. Furthermore, Cameron and Christie found that on completion of their training health visitors’ practice is closely aligned to the traditional health visiting role with its focus on mothers and children [35]. These studies justify the call for nursing curricula to focus on social justice, in order to prepare nurses to address fundamental determinants of health inequalities. The outcomes of these studies justify the call for nursing curricula focus on social justice to prepare future nurses to address fundamental determinants of the inequalities in health. However, not much has been published about public health nurse educators’ (PHNE) understanding of public health principles of social justice and health inequalities.

This article examined PHNE’s understanding of public health as a strategy to reduce health inequalities. It is the second article from the project which investigated the public health nurse educators’ knowledge of public health [15]. Mabhala [15] took an overview of three essential themes emerged from the findings that describe lecturers’ understanding of public health teaching. The current paper focuses on PHNEs’ conceptualisation of public health as a strategy to reduce health inequalities. The main contribution of this paper is that for the first time as far as the researcher’s knowledge is concern it offers a model to examine the dimensions of health inequalities.

### Study design and methods

The design of this study was influenced by Charmaz’s [41-43] constructivist grounded theory (CGT). The stages of data collection and analysis also drew heavily on other variants of grounded theory, including those of Glaser and Strauss [44], and Strauss and Corbin [41,43].

A total of 26 individual semi-structured interviews were conducted with the eleven participants. The sample comprised of educators with extensive knowledge and experience of public health practice and higher education teaching. They were all directly involved with public health curriculum development and teaching. Table 1 illustrates participant’s clinical background and cumulative experiences in clinical practice and higher education.

**Table 1:** **Participants’ background and their cumulative experiences**

**Clinical background**	**Number of participants**	**Cumulative years in clinical practice**	**Cumulative years in higher education teaching**
Health visiting	3	42	37
School nursing	1	30	10
District nursing	1	20	12
Occupational health	2	32	14
Mental health	1	15	20
General nursing	2	17	25
Learning disability	1	15	25

Consistent with CGT there was an iteration between analysis and data collection; this meant that the investigator had to determine the sources of data and/or which participants were likely to provide the rich data needed for category development [45-48].

Three sampling strategies were used: purposive, criterion and theoretical. Purposive and criterion sampling was used to generate themes for further exploration. The interview schedule for the first phase consisted of 10 questions such as:

1. Before we start can I ask you to tell me about your own history and experience in nurse education e.g. when did you come into nurse education, what is your own nursing background?
2. Can you talk about the areas of public health that you teach?
3. What are the ‘big ideas’ that underpin public health nurse education for you? What is public health about?

Theoretical sampling was undertaken in accordance with Strauss and Corbin’s [45] recommendation that the filling in of poorly developed categories be done through review of memos or raw data, looking for data that might have been overlooked [47], and returning to key participants asking them to give more information on categories that seemed central to the emerging theory [48]. The questions asked at theoretical sampling stage were guided by the analysis, and included questions such as:

‘In your earlier interview you mentioned that that your perception of public health is influenced by your personal life.

1. Could you elaborate on how your personal life including family, community, social life, schooling and work experiences influence your understanding and interpretation of public health?
2. Could you talk about incidents in your professional life that explain your perception of public health?’

The first published article from this project [15] provides a detailed account of methodological underpinning, methods of data collection, analysis, and ethical consideration in previous article from this project.

## Results

PHNEs identified three areas of public health that constitute the structure of the public health curriculum framework: ‘health inequalities [as] the main thing’; socio-economic determinants in health are the ‘facets of life’; and that nurses should ‘engage with policy and politics’.

### Health inequalities is the main thing

Tackling health inequalities was one of the most frequently recurring themes. Two distinct views emerged: some proposed population approaches focusing on upstream preventive strategies, whilst others proposed behavioural approaches focusing on empowering vulnerable individuals to improve their own health. PHNE 4, for example, cited the first approach:

*Health inequalities is the main thing, epidemiology is another one, it’s identifying where health issues are, where they come from and what the causes of them are and you can only do that by studying the population group looking at the epidemiology of the population, identifying the strategies perhaps to try and prevent ill health by working upstream by sort of putting together preventive strategies and I think in specialist practice programme with variety of students some do work in the primary prevention and other students and I think district nurses work with clients groups who have after-effects of years of ill health.* [PHNE 4]

Two significant points emerged from this extract: first, her main interest was epidemiology, and she was motivated by her desire to acquire scientific evidence to explain the cause of the health inequalities that she witnessed first-hand as a district nurse. Second, as a health professional she felt that her individual interventions were inadequate for addressing the problems facing the local population, and proposed that upstream strategies were appropriate approaches.

She suggested that epidemiology provided skills to identify or specify the nature of the problem. She recalled her experience as a district nurse:

*Health inequalities is an area, it’s something that I can link in very much to practice when I was working in district nursing because the area that I work geographically was an area of high deprivation. Although I knew that anyway when I was working in practice the public health information all the data, all epidemiology that give you the context, gives the background evidence to support all that really it all links together now, so that is what I teach the students where to find the information, what are the causes of ill health and what do they think are the causes of ill health. It is interesting at the moment because we have students from hospital they are doing health inequalities now and it’s interesting to hear their perspectives of inequalities because they have not worked in the community before, it’s good to see what they think are the main causes of ill health and that somebody’s health is affected by 70% of what happens outside the NHS.* [PHNE 4]

The idea of tackling fundamental causes of health inequalities was also expressed by PHNE 1:

*We can educate people about health but unless inequalities on things such as education, environmental issues, if they aren’t addressed then we will have a very limited impact.* [PHNE 1]

PHNE 5 recalls the critical moments that changed his views about individual interventions:

*Yes I read a report it was part of Whitehall studies, it was report into pensionable ages of the prison civil service staff, it reported it was looking at the simple fact that the average prison officer on retirement only lives for six months post retirement and die …This opened my eyes into all sorts of the inequalities in health that I never thought of … think of the inequalities and poor health, raised my awareness of the failures of the NHS individual approach to health and awareness of the wider political agenda.* [PHNE 5]

The extract below gives a different perspective on tackling health inequalities. PHNE 3 emphasises tackling differences in access to healthcare as opposed to health; she also focuses on the individual behavioural issues that restrict people from accessing healthcare services, and empowerment of individuals by providing information to enable them to make healthier choices:

*I think that if [students] have a grasp of what inequalities in health might be, I’m hoping that they might be able to identify individuals as well as groups that fit into those categories, subsequent when it comes to them having to give actual information or being on board with any kind of advice they have some kind of understanding of why people behave the way they do. It is very much on the individualistic basis underpinned by some of the theories that we actually trying to give them. And also although we’re not political animals, but if they buy into any political agenda, they see what the government is trying to do. It’s very much now trying to get individuals to make those changes. So from the nursing students’ point of view they have areas where they can make some impression on people.*

*… I guess vulnerable areas; vulnerable groups that may arguably not have the same kind of information, don’t feel empowered perhaps to access healthcare. Maybe they don’t even know it is there and therefore you can’t avoid this huge focus on health inequalities as well. They are my big things I think. If you can get them to understand what we mean by public health, if you can get them to understand what we mean by those element like I said, the determinants of health and health inequalities what they mean.’* [PHNE 3]

The last part of this comment could be interpreted as meaning that health inequalities affect vulnerable groups who are either not empowered to access health services or don’t know where they are; and therefore if you empower them with the knowledge and confidence to access health services, you reduce some of the inequalities.

The notion of tackling health inequalities through motivating individuals to change behaviour was also cited by other participants including PHNEs 1, 6, 8 and 9. Amongst the concepts that characterised PHNE 8’s approaches to public health teaching were advocacy, personal responsibility, vulnerability, and providing information to enable vulnerable and disempowered groups to make healthier personal choices. All seemed to promote behavioural approaches to health inequalities:

*I think the major role that I certainly advocate with the public health community specialist practice is advocacy. I think advocacy is the one that have been neglected in the new public health arena where we are very much about giving people the responsibility for their own health and in actual fact they have neither skill or motivation to take on that role so we need advocacy.*

*I think health is a personal choice but is a choice with caveat, you can only make choice if you are informed and also if are in that arena in the circle of change that actually enables you to make that change you need to make that where advocacy comes in we all know from our experiences for example smoking cessation that people fail several times and failure reinforces that belief that they cannot achieve the cessation position but in actual fact with advocacy and support and information we can move people to that point.*

*I think knowledge is power if given in a continuous drip feed, consistently at appropriate level across the life span. I think advocacy is very important because all people are vulnerable at some stage in their lives, some people move into the stage of vulnerability and move out of it very quickly and others live in almost permanent state of vulnerability. And it is the vulnerable that suffer the greatest inequalities in health and therefore if we lose vision to advocacy, we lose power to help these people to move on from that state of vulnerability.* [PHNE 8]

PHNE 9 maintained that access to healthcare provision was a major issue in the UK:

*There are issues around the access to healthcare even in the UK where there is supposed to be universal access to health the issues of inequalities go beyond that, it’s about fairness; I mean …working with these asylum seekers who are not registered yet, apparently once registered they become refugees, their status changes, but while they’re asylum seekers they have got no right to access healthcare, where is fairness in that?*

*…citing an incident of a lady who came from Africa who was poorly when she came, she was wrongly diagnosed with AIDS when she was in Africa because apparently when you’re ill in her country that was the first thing that came to people’s mind, but when she went to the doctor she was diagnosed with type 1 diabetes mellitus (DM).* [PHNE 9]

It is clear from the above extract that for these PHNE inequalities in health is about fairness. This participant identified the population group at risk of inequalities in health due to their immigration status; and proposes that access to healthcare service should be a human right that transcends individual social status.

### Socioeconomic determinants of health

Participants in this study frequently argued that public health affects or is affected by all ‘facets of life’; it emerged that they were referring to the socio-economic determinants of health (SEDH). This was first made explicit by PHNE 6 who made reference to Dahlgren and Whitehead’s model:

*Well we all know socioeconomic determinants of health model… it’s a bible really isn’t it? All people are familiar with Dahlgren and Whitehead’s model really… because the layers identified there have a great impact on health; take out any these the facets of life collapses.* [PHNE 6]

‘…It’s a bible really’ reflects the value attached to this model. All PHNEs in this study regarded SEDH as an important component of the structure of the public health curriculum, referring to the whole range of processes through which social factors impact on health:

*Public health [is affected by] all facets of life whether it’s just day to day living, employment, where you live, how much money you’ve got, your social life, cultural political aspect, all impinge on health.* [PHNE 1]

However, SEDH were not understood or made explicit by all PHNEs in the same way; rather, it depended on their discipline or relationship with the public health field. For example, PHNE 11 offered an insightful account into how the theory of SEDH can explain health inequalities in a mental health context. He explained that people with mental health problems shared a high burden of all determinants of health inequalities compared to the general population: they tended to have poor education, and thus end up unemployed or in low paid employment, and have disproportionately high prevalence of lifestyle related conditions such as cardiovascular disease, obesity and smoking related disease. These conditions could be attributed to a combination of a poorly paid job and poor education, which in turn resulted in them having limited choices in terms of access to commodities essential for good health such as diet, exercise and health literacy. His understanding of the application of SEDH in a mental health context related to his experience as a mental health nurse:

For the whole of my career I have seen the inequalities in health… every mental health nurse with… brain has… this cliché about people’s choice is a lot of ***… excuse my French. Look at unemployment for example… we know that people with mental health have highest level of unemployment, highest smokers, highest in obesity and if luck to have job lowest paid. What choices do they have? [PHNE 11]

Others such as PHNE 1, 2 and 10 came from a hospital-based nursing background, and perceived the SEDH as a holistic approach to assessment of patient conditions such as cardiac or respiratory disease. They proposed that consideration of the role of socioeconomic circumstances in disease development helped them gain a holistic view of their clients’ nursing care. Both the following quotations illustrate how participants proposed this model could be applied in a hospital setting:

*Public health affects all aspects of people’s lives whether it’s just day to day living, employment, where you live, how much money you’ve got, your social life, cultural, political aspect, all impinge on health. If you are a hospital-based nurse the best way to look at it is to think of someone coming to the hospital with coronary heart disease, what you have to ask yourself is: what circumstances got them there in the first place, looking in the facets, public health facets, lifestyle issues, deprivation, social, political issues which actually got them to the situation they are in. Because sometimes students think that certain public health issues sit outside their remit, for example they may think well what housing has anything to do with what I do, I work in the hospital.* [PHNE 1]

This view about the relevance of SEDH to other aspects of nurses’ practice is shared by most participants:

*I think for example in holistic care you might be looking at respiratory condition or a cardiac condition. For me you have to consider all the socioeconomic and political factors because the medical model would say we bring people in, we treat them and we let them go home. From a more holistic point of view it is looking at all socioeconomic factors that contributed to that disease process in the first place.* [PHNE 2]

Participants who came from health promotion backgrounds proposed application of SEDH in their health promotion nurse's role to determine why individuals chose to engage in unhealthy lifestyles, arguing that effective approaches to health promotion and behavioural change interventions involved consideration of socioeconomic and political/environmental factors that might influence people’s attitudes towards health. These concepts emerged from the interview with PHNE 6, who stated:

*Public health is an intervention to change behaviour.* [PHNE 6]

When PHNE 6 was asked to talk about the ‘big ideas’ that underpin public health nurse education, her response was:

*I suppose really it’s understanding of the wider determinants of health I think that is really key, because when I sort of went into public health, health visiting helped me develop understanding that you cannot apply the same principle to every situation, everybody has individual needs, I think for students you can say okay you have a key health promotion message but how you approach that with an understanding of where people are coming from. I think as nurses I think in the past we are sort professional judgemental really, in that we sort of think we know best what they [patients] should be doing that. I’m quite new to teaching but having that opportunity to influence the students so that they realise that it’s not necessarily that person’s fault, there are lot of reasons why they choose that behaviour and I think for the short time I have been here it’s nice to see the students [develop] that understanding; therefore less judgemental about why people smoke, why they drink, why they have unprotected sex and things like that.* [PHNE 6]

Expressions such as ‘individual needs’, ‘key health promotion message’ and ‘choose that behaviour’ in this excerpt gives a distinct sense of the individual behavioural approach to tackling health inequalities. The same sense was evident in PHNE 3’s comment:

*I think that if they have a grasp of what inequalities in health might be, I’m hoping that they might be able to identify individuals as well as groups that fit into those categories, subsequent when it comes to them having to give actually information or being on board with any kind of advice, they have some kind of understanding of why people behave the way they do. It’s very much on the individualistic basis underpinned by some of the theories that we’re actually trying to give them.* [PHNE 3]

Again, one got a sense that this PHNE believed in promoting behavioural change as a way of reducing health inequalities. She believed that understanding of SEDH was important to enable nurses to tailor their health promotion messages according to individual needs.

### Engage with policy and politics

It was evident from participants’ comments that they regarded understanding of policy and political influences on public health as essential for students. The UK policy directives most frequently cited included *Choosing Health: Making Healthy Choice Easier*, the Wanless reports and the Darzi report. Participants expressed different views about what understanding of government policy and politics meant in relation to nurses’ roles. PHNEs who believed that tackling health inequalities required tackling their fundamental causes tended to suggest that the nurses’ role should involve engagement with policy and politics. They furthermore suggested that for nurses to be effective contributors to tackling health inequalities, they needed to develop an understanding of policy processes and increase their political engagement: for overall population (not just individual) improvement, nurses needed to affect the policy. PHNE 2, a proponent of this approach, argued that as long as the conditions that introduced differences in burdens and benefits remained intact, efforts to reduce inequalities would not be successful; the nurses’ role was to gain understanding of political parties’ ideologies and their positions on health, present the health argument for policy changes, and take part in the political process in the form of voting for those that are making inroads into addressing health inequalities. This is how PHNE 2 expressed this view:

*I know I have to be apolitical when I’m talking about politics so not to influence people, but student nurses are citizens of the country and also nurses. I think you have to engage with policy and politics because otherwise from the health point of view I don’t think we are going to see any great changes, because any changes have to be supported by the government. We can educate people about health but unless inequalities on things such as education, environmental issues, if they aren’t addressed, then we will have a very limited impact. It has to be holistic and has to be global as well, so I think when I teach a subject it’s about thinking how you can draw into that situation appropriate public health messages, because Wanless Report put emphasis on us nurses being out there being role models.* [PHNE 2]

PHNEs in this study expressed contrasting views about the potential of specific policy directives to reduce health inequalities. These views were particularly evident in their analysis of the two key principles underpinning *Choosing Health*: choice and personal responsibility. In relation to the principle of ‘choice’, PHNE 2 said:

*I think ultimately people do have choices, we are autonomous beings, but I think there is a ceiling in those choices in as much as I can decide to eat healthily, I can decide to do the best that I can to eat a healthy diet, then there comes a ceiling as to what choices are made available to me within my environment, what the government is doing in terms of food legislation which is something I have been grappling with for a long time. I think the people have to take responsibility and have to act as autonomously as possible. If you are not happy, we are a democratic country and one of the most powerful instruments for change we have as citizen of the country is a right to vote; so therefore we should be voicing our concerns and voting or lobbying parties to make inroads into areas where we feel we don’t have much control over.* [PHNE 2]

PHNEs 2 and 5 felt that the principles that underpin *Choosing Health* and subsequent policy documents which promote choice served to exacerbate health inequalities, as the only beneficiaries of this were affluent groups. For example, PHNEs 2 and 5 criticised *Choosing Health* for promoting personal responsibility and choice as a way of tackling health inequalities, when people who suffer their worst effects were those who neither had choice nor were empowered to take personal responsibility. PHNE 5 stated that:

*Taking that forward to public health, the* Choosing Health *document that says we must give people more responsibility, we must work in partnership, how can get them to take responsibility if there so much on their way? How can we get partnership working between the haves and disempowered people?* [PHNE 5]

PHNE 8 criticised the government’s notion of choice and personal responsibility:

*I think advocacy is the one that have been neglected in the new public health arena, we gone down the path of giving people the responsibility for their own health and in actual fact they have neither skill or motivation to take on that role, so we need advocacy.* [PHNE 8]

PHNEs 1, 3 and 6 expressed positive views about the direction of UK policy, feeling that *Choosing Health* provided a direction and guide for nurses’ practice. PHNE 1 was positive about the government position regarding reducing health inequalities, stating that:

*I think the government policy* [Choosing Health] *is certainly going in the right direction, certainly highlighting the relevant issues, but addressing them is often difficult to put them into practice if there are insufficient resources or those resources are not targeted effectively.* [PHNE 1]

PHNE 6’s narrative provided insight into the group of participants who felt that *Choosing Health* provided a guide to their practice:

*I suppose really I sort of fall into health promotion more, so probably a lot of that is due to my background as a health visitor as health visiting is very much about promoting health… the key policy to me is* Choosing Health*, that sort of set the ground… that focus very much on promoting public health. The other thing is the whole notion of early education is ultimately what I sort of hope to empower people to realise that getting there early, parents and education is key if will make a difference.* [PHNE 6]

This view was also shared by PHNE 3:

*I think if I think about health education and policy I guess the one that we’re certainly working towards is* Choosing Health*, what it does I suppose in many ways it encapsulated some of the major public health issues, and that allows us therefore to go away and examine them in a much broader way if you like, from those six or so main targets if you like in terms of public health there are various policies that sit within those different areas as well – healthy lives, healthy weight etc.* [PHNE 3]

However, it was evident that PHNE 3 was concerned about the availability of resources to put these guidelines into practice. Like PHNE 1, PHNE 9 also expressed concerns that government policy decisions were influenced by resources rather than needs. This view was made explicit by PHNE 9:

*Finance is the big driver and the target is the obvious driver and another thing really is that the government is only in power for five years, they have no really aspirations to make long term changes because there is so much self-interest with them when you look at them they will always a quick fix.* [PHNE 9]

The issue of financial influence on policy decisions was expressed by other participants; for example, PHNE 3 stated:

*I think would be something around finances, it would be around the ability to make changes, whether there are responsibilities and I guess is what is highlighted on their agendas as well. What is important for that particular party at that time? The political ideologies of the day. Certainly finances because it has a knock on effect on what care delivery is required. Arguably I guess as nurses our influence on policy development is getting less and less particular because fewer nurses are being employed different skill mixes then you’ve got different levels of understanding and ability to make difference. I think the government thinks we have ability to influence but it’s just about having resources to enable us to deliver those changes.* [PHNE 3]

## Discussion

All participants described health inequalities as ‘the main thing’ [15]. They further identified epidemiology, SEDH, and policy and politics as the components that shape their understanding of public health strategies to reduce the health inequalities [15]. The synthesis of the current study findings and data from previous studies informed the development of a conceptual framework (see Figure 1) that describes three dimensions – science, ethics and human rights – that frame the debate about using social justice principles as a foundation for strategies to reduce health inequalities [15].

The first excerpt from PHNE 4 articulates the function of epidemiology in relation to health inequalities. It emerged from this study that understanding of the science of public health in the form of epidemiology enables us to establish how health inequalities are created and sustained. This view is consistent with the conceptual framework (Figure 1) which sees epidemiology and an essential tool to establish existence, the extent and the effectiveness of the measure to reduce health inequalities. Based on this evidence and several studies [49-51], Mabhala [14] described epidemiology as a science discipline driven by moral and ethical concerns about the injustice of health inequalities. This description resonates with Venkatapuram and Marmot’s [51] summation that if one assumes that the ill health of individuals is an important moral concern, then it stands to reason that following through on that concern is what drives the pursuit of scientific knowledge of the causes, distribution patterns, and consequences of ill health. They go on to assert that such concern also motivates identifying and implementing appropriate social interventions to address socially determined ill health [50,51]. This evidence informed the proposed ethical and moral dimension in our conceptual framework of the inequalities in health (Figure 1).

Mabhala [14] went further to argue that situating debate about health inequalities within science and ethical dimensions is insufficient. He drew upon the Alma-Ata Declaration, which confirms health is a human right, to propose *human right dimension* of conceptual framework, which, argues that where it is possible to provide social goods essential for the support of health and well-being and these are not being provided, then that is a human rights issue [14]. Arguably, promoting health equality as a human right has the potential to conscientise society so that claims for health equality receive adequate public and political expression, rather than it being seen as an abstract concept understood only by academics and politically subversive groups [13].

Two contrasting views on how inequalities could be tackled emerged in this study. Some proposed a population approach focusing on upstream preventive strategies, whilst others proposed behavioural approaches, focusing on empowering vulnerable individuals to improve their own health. The latter view is consistent with evidence that despite upstream interventions to reduce health inequalities having demonstrably more leverage than individual behavioural interventions [12,20,24,31,33,34], nurses are likely to use individual-family level interventions rather than population approaches [35]. In the UK it has been found that contrary to the previous Labour governments’ endorsement of the public health role of nurses [52], they spend a substantial proportion (61%) of their time on individual behavioural interventions [36-38]. Arguably, the promotion of these as a strategy to reduce health inequalities is inspired by policies produced following *Securing Good Health for the Whole Population* [53], which put greater emphasis on promoting choice and lifestyle behavioural change than on upstream interventions focusing on fundamental determinants [9,53-57].

PHNEs in this study identified SEDH as an appropriate model to describe how public health affects and is affected by all facets of life. The contrasting views between those who favour individual behavioural approaches and those who favour upstream population approaches were also evident in PHNEs’ descriptions of SEDH and their understanding of policy. Some described policy in terms of proactive approaches, illustrating how nursing as a collective body can influence the shape and implementation of specific policy areas. Others tended to take a reactive approach that seeks to describe the consequences of policy development upon nursing [58-60]. This was particularly evident in their description of *Choosing Health* – while some took a critical approach to its principles, others tried to identify specific and precise policy statements on what nurses do or are required to do [58]. Arguably, these conflicting views signify uncertainty about the position of the nursing profession in relation to the principles guiding policy makers.

## Conclusion

Despite the proven effectiveness of upstream interventions at reducing health inequalities, some nurses are better equipped to perform individual behavioural interventions than take population approaches. Some PHNEs described policy in terms of proactive approaches, illustrating how nurses can collectively influence the shape and implementation of specific policy areas. Others tended to take a reactive approach that seeks to describe the consequences of policy development upon nursing.

### Limitations

Because participants were recruited from one faculty in a relatively mid-size university, one limitation of this study was the representativeness of the sample. Public health educators (academics) come from a wide range of backgrounds; the sample in this study consisted of one group of public health educators [1], and in a larger population that included other disciplines the results might be different. The investigator was known to some of the participants; though every effort was made to account for respondents’ bias, this can be seen as a potential limitation of this study. It has to be acknowledged that the method of recruitment of the 11 participants generates a bias in favour of those with a particular interest in public health. The methodology used in this study (CGT) advocates mutual construction of knowledge, so the researcher understands and interpretations may have had some influence in the research process as the researcher is an integral part of the data collection and analysis.

The researcher had a benefit of ‘insider’ status, having worked within the field of education and public health for more than twelve years; I had an in-depth knowledge of public health and experience of being a public health educator that was used to enhance the quality of the interview process [61]. Furthermore, ‘insiderness’ meant I had privileged access, familiarity, and rapport with the study participants [62,63]. This privileged access to participants also created greater flexibility with regard to interview times and cost-effectiveness in the sense that there were no travel costs
